# Targeting mTOR for Anti-Aging and Anti-Cancer Therapy

**DOI:** 10.3390/molecules28073157

**Published:** 2023-04-01

**Authors:** Wencheng Fu, Geng Wu

**Affiliations:** State Key Laboratory of Microbial Metabolism, School of Life Sciences & Biotechnology, the Joint International Research Laboratory of Metabolic & Developmental Sciences MOE, Shanghai Jiao Tong University, Shanghai 200240, China

**Keywords:** senescence, aging, anti-aging, cancer, anti-cancer therapy, mTOR, mTOR inhibition

## Abstract

The balance between anabolism and catabolism is disrupted with aging, with the rate of anabolism being faster than that of catabolism. Therefore, mTOR, whose major function is to enhance anabolism and inhibit catabolism, has become a potential target of inhibition for anti-aging therapy. Interestingly, it was found that the downregulation of the mTOR signaling pathway had a lifespan-extending effect resembling calorie restriction. In addition, the mTOR signaling pathway promotes cell proliferation and has been regarded as a potential anti-cancer target. Rapamycin and rapalogs, such as everolimus, have proven to be effective in preventing certain tumor growth. Here, we reviewed the basic knowledge of mTOR signaling, including both mTORC1 and mTORC2. Then, for anti-aging, we cited a lot of evidence to discuss the role of targeting mTOR and its anti-aging mechanism. For cancer therapy, we also discussed the role of mTOR signaling in different types of cancers, including idiopathic pulmonary fibrosis, tumor immunity, etc. In short, we discussed the research progress and both the advantages and disadvantages of targeting mTOR in anti-aging and anti-cancer therapy. Hopefully, this review may promote more ideas to be generated for developing inhibitors of mTOR signaling to fight cancer and extend lifespan.

## 1. Introduction

**Figure 1:**
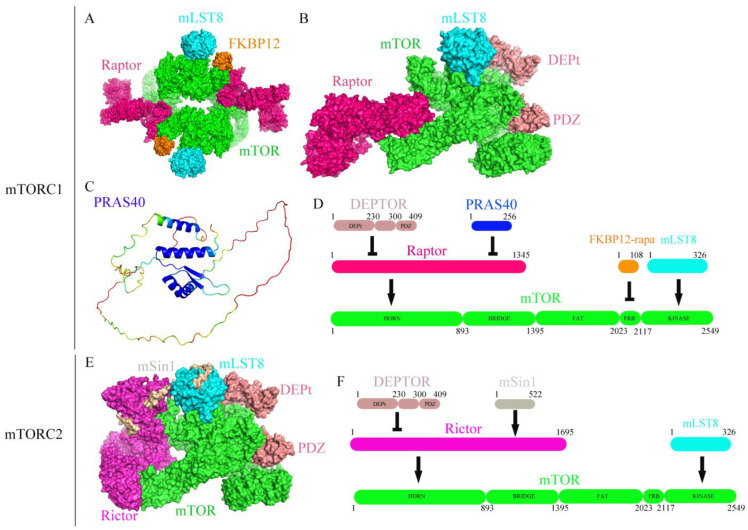
mTORC1 and mTORC2. (**A**) The structure of human mTOR complex 1 (mTORC1); (**B**) the structure of DEPTOR bound to human mTORC1; (**C**) the Alphafold2-predicted structure of PRAS40; (**D**) mTORC1 subunits and their respective binding sites on mTOR; (**E**) the structure of DEPTOR bound human mTOR complex 2 (mTORC2); (**F**) mTORC2 subunits and their respective binding sites on mTOR.

The mammalian target of rapamycin (mTOR, also known as FRAP or SKS) is a phosphatidylinositol 3 kinase-related protein kinase (PIKK) that controls cell growth, metabolism, survival, immune responses and so on. mTOR forms two complexes: mTOR complex 1 (mTORC1) and mTOR complex 2 (mTORC2). mTORC1 consists of six subunits (Figure 1A–D): mTOR (including Horn, Bridge, FAT, FRB and the kinase domains), raptor (regulatory protein associated with mTOR), mLST8 (mammalian lethal with Sec13 protein 8, also known as GBL or WAT1), PRAS40 (proline-rich Akt substrate of 40 kDa), DEPTOR (containing the PDZ domain and the DEP domain tandem, DEPt), and FKBP12, which binds to the FRB domain of mTOR (Figure 1C). On the other hand, mTORC2 consists of mTOR, rictor (rapamycin-insensitive companion of mTOR), mSin1, mLST8, and DEPTOR (Figure 1E,F). The structures and functions of mTORC1 and mTORC2 are conserved in eukaryotes, such as yeast, worms, flies, and mammals. Raptor promotes the recruitment of substrates to mTORC1 by binding to the TOR signaling (TOS) group on the substrates. mLST8 may stabilize the kinase activation loop of mTOR. PRAS40 is a raptor-interacting protein and inhibits mTORC1. DEPTOR, which is an mTOR-binding protein, can inhibit both mTORC1 and mTORC2. mSin1 is critical for the insulin-dependent regulation of mTORC2 activity, with its PH domain inhibiting the catalytic activity of mTORC2 in the absence of insulin. mTORC2 can modulate the phosphorylation of protein kinase Cα and the actin cytoskeleton. mTORC1, but not mTORC2, is inhibited by the rapamycin-FKBP12 complex. In contrast, mTORC2 is insensitive to rapamycin treatment.

The major functions of mTORC1 are to promote anabolism and to inhibit catabolism. mTORC1 promotes protein synthesis by phosphorylating p70 S6 kinase 1 (S6K1) and eIF4E-binding protein (4EBP). mTORC1 also promotes lipid synthesis via the sterol response element binding protein (SREBP) transcription factor. Meanwhile, mTORC1 can promote the synthesis of nucleotides by the ATF4-dependent expression of methylene-tetrahydrofolate dehydrogenase 2 (MTHFD2), and S6K1-dependent activated carbamoyl-phosphate synthetase (CAD). Additionally, mTORC1 can also promote a shift in glucose metabolism by increasing the translation of the transcription factor HIF1α. mTORC1 regulates cell growth and metabolism, while mTORC2 mainly controls proliferation and survival by phosphorylating certain members of the AGC family of protein kinases, including PKA, PKG and PKC. Compared with mTORC1, mTORC2 mainly acts as an effector of insulin/PI3K signaling. Vellai et al. first revealed the key role of mTOR in aging in 2003 [1]. In recent decades, lifespan extension by inhibiting the mTOR pathway has been proved by more and more evidence. In addition, the activity of mTOR is also frequently deregulated in many kinds of human cancers, such as breast, lung, liver, prostate, renal and pancreatic cancers. Statistical data show that advanced age is a very important risk factor for cancer, and cancer incidence steadily increases with age [2]. This review describes the research process and future direction of mTORC1 inhibition in anti-aging and cancer therapy.

## 2. Anti-Aging by Targeting mTOR

Apparently, the mTOR signaling pathway is a key anti-aging target, and there has been much supporting evidence. Mice with two hypomorphic (mTOR△/△) alleles, 129S1 and C57BL/6Ncr, expressed mTOR at approximately 25% of the wild-type levels and exhibited an approximately 20% increase in median survival through reduced mTORC1 and mTORC2 activity [3]. Even though the mTOR△/△ mice were smaller than the wild-type mice, they did not show any difference in food intake, glucose homeostasis, or metabolic rate. Consistent with their increased lifespan, mTOR△/△ mice exhibited a reduction in a number of aging tissue biomarkers. Attenuated mTORC1 signaling is sufficient to extend lifespan independent of alterations in glucose homeostasis, as in female mice, but not male mice, heterozygous in mTOR or mLST8 (*mtor*^+/−^ or *mlst8*^+/−^) exhibited reduced mTORC1 activity and prolonged lifespan but still maintained normal glucose tolerance and insulin sensitivity [4]. In female mice, but not male mice, the deletion of *s6k1* leads to increased lifespan and resistance to age-related diseases, such as bone diseases, immune system dysfunction, and a loss of insulin sensitivity [5]. The deletion of *s6k1* induced gene expression patterns similar to those seen in caloric restriction (CR) or those with pharmacological activation of adenosine monophosphate (AMP)-activated protein kinase (AMPK), which is a conserved metabolic regulator in response to CR [5]. The lifespan of *tsc1*-overexpressed female mice, but not male mice, was significantly increased. A modest increase in TSC1 overexpression can enhance overall health, especially cardiovascular health, and improve female survival [6]. Garratt et al. found that only males with acarbose and 17-α estradiol treatment obtained lifespan extension. They ascribed male-specific metabolic health and survival to be associated with hepatic mTORC2 signaling, Akt activities, and FOXO1a changes [7].

Although it seems that the downregulation of mTOR (including both mTORC1 and mTORC2) can result in anti-aging, its mechanism is still not clear. In the above, we have mentioned that anti-aging through the inhibition of mTOR was similar to caloric restriction in some aspects. Kaeberlein et al. found that the dietary restriction of *tor1*△ or *sch9*△ failed to increase lifespan in the budding yeast *Saccharomyces cerevisiae* [8]. This result suggests that the benefits of dietary restriction and the downregulation of mTORC1 at least partially overlap [9]. However, several studies also demonstrated TOR-independent longevity activated by dietary restriction. What is more, rapamycin can extend the lifespan beyond the maximum level induced by dietary restriction. Rapamycin is specific for inhibiting mTORC1, and thus it enables us to investigate mTOR’s anti-aging mechanism using rapamycin. Bjedov et al. showed that feeding rapamycin to adult *Drosophila* extended the lifespan through the downregulation of S6K activity and the upregulation of 4E-BP1 activity [10]. In addition to lifespan extension, rapamycin also increases stress resistance and reduces fecundity. Rapamycin mediates lifespan extension independent of insulin/Igf signaling (IIS) and AMPK. In addition, rapamycin also promotes autophagy and decreases protein translation. Autophagy is activated under nutrient-limited conditions and is inhibited by mTOR activity through the inhibitory phosphorylation of autophagy-initiating kinases ULK1 (ATG1) [11], ULK2, ATG13, FIP200 [12], and lysosome-localized TFEB [13]. Lopez-Otin et al. enumerate nine tentative hallmarks that represent common denominators of aging in different organisms, including genomic instability, telomere attrition, epigenetic alterations, loss of proteostasis, deregulated nutrient sensing, cellular senescence, mitochondrial dysfunction, stem cell exhaustion, and altered intercellular communication [14].

Residual senescent cells contribute to aging-related diseases [15]; therefore, it will be helpful for us to understand aging through senescent cells. van Vliet et al. showed that AMPK-mediated mTOR downregulation can inhibit the senescence-associated secretory phenotype (SASP) [16]. Demaria et al. demonstrated that the senescence of non-tumor cells could help tumor growth and metastasis, and they showed that eliminating senescent cells could reduce chemotherapy side effects, such as bone marrow suppression, cardiac dysfunction, cancer recurrence, and fatigue [17]. These results suggested that reducing SASP may contribute to reducing chemotherapy side effects and extending lifespan. Baker et al. found that clearing senescent cells improves aging-related phenotypes [18,19]. Carroll et al. found that mTORC1 can be constitutively active and resists serum and amino acid starvation due to stress, replication failure, or oncogene activation in senescent human fibroblasts [20]. They thought that this was caused in part by the depolarization of the plasma membrane of senescent cells, which leads to defects in primary cilia and failure to suppress growth factor signaling. Furthermore, increased autophagy and high levels of intracellular amino acids may support mTORC1 activity under starvation conditions. Hence, they suggested that the inhibition of mTORC1 can contribute to the cleaning of senescent cells and delaying aging. Walters et al. showed that the acute short-term treatment of human skin fibroblasts with low-dose ATP-mimicking pan-mTORC inhibitor AZD8055 reversed many of the phenotypes that developed as cells approached replicative senescence, including reduced cell size and granularity, the loss of SA-β-gal staining, and the regaining spindle-shaped morphology of fibroblasts [21]. AZD8055 treatment also induced rearrangements of the actin cytoskeleton, which provided a possible explanation for the observed rejuvenation. Their finding suggests that the combined inhibition of mTORC1 and mTORC2 may be a promising strategy to reverse the development of senescence-associated traits in near-senescent cells. It was reported that a mild restriction of cytoplasmic protein synthesis abolished replicative senescence in virtually all cell types and extended the lifespan in *C. elegans* [20]. Pan et al. found that overexpressed Yes-associated protein (YAP), which is a major effector of the Hippo signaling pathway, could increase senescence in both HUVECs and vascular tissues following autophagic flux blockage and mTOR pathway activation [22]. Furthermore, mTOR inhibition could relieve YAP-promoted cellular and vascular senescence.

Recent studies have shown that a reduction in nicotinamide adenine dinucleotide (NAD^+^) is a key contributor to aging-related metabolic decline. Tarrago et al. revealed that a thiazoloquin(az)olin(on)e CD38 inhibitor, 78c, increased NAD^+^ levels and activated pro-longevity and health span-related factors, including sirtuins, AMPK, and PARPs, and they observed the inhibition of pathways, including mTOR-S6K, ERK, and telomere-associated DNA damage [23].

mTOR signaling is constitutively active during senescence, which is caused by replication failure, oncogene activation, and other stresses [20], and it may drive the geroconversion process, i.e., the transition from proliferation to senescence without the inhibition of growth [24,25]. The gradual decline in mitochondrial efficiency is an important hallmark of aging. Senescent cells accumulated chronic DNA damage and their mitochondria became dysfunctional, with reduced oxidative phosphorylation efficiency and increased reactive oxygen species production [26,27,28]. mTOR provides a critical link between the cell’s energy balance and mitochondrial loading and regulates mitochondrial biogenesis (including PGC-1-β-dependent mitochondrial biogenesis and translation of mRNAs via 4EBP1 inhibition, with mitochondrial oxidative function controlled through the YY1-PGC-1α mechanism) [29,30] and mitophagy [31,32].

Damage-responsive ATM/ATR kinases phosphorylate and activate mTORC2, which then phosphorylates Chk1, leading to a proliferation arrest in the S phase or the G2/M phase. mTORC2 is associated with this arrest in breast cancer cells [33]. The mTOR/S6K pathway is also regulated by p38α MAPK and p53 [34]. Xie et al. found that the mTOR-S6K pathway led to the phosphorylation of the Ser60 site of RNF168, inhibited its E3 ligase activity, accelerated its proteolysis, and weakened its function in DNA damage response, leading to the accumulation of unrepaired DNA and genome instability [35].

Lipid metabolism disorder results in deregulated triglycerides and cholesterol and can induce senescent cells [36]. mTORC1 signaling activates SREBP transcription factors to drive fatty acid biosynthesis for lipogenesis [37] and regulates 4EBP1, 4EBP2, PPARγ, PPARα, and PGC1α activity, which in turn regulates fatty acid oxidation [38,39,40,41]. As for immune function, Hurez et al. found that chronic mTOR inhibition of mice with rapamycin feeding altered T cells, B cells, myeloid and innate lymphoid cells and gut microbiota, and extended lifespan in immunodeficient mice [42]. Mannick et al. also found that the selective inhibition of TORC1 had the potential to improve immune function and reduce infection in elderly adults [43]. These data suggest that mTOR inhibition induces a crossover network in multiple ways to contribute to anti-aging.

## 3. The Complexity of Anti-Aging by Targeting mTOR

From the above, we can see that the mechanism of anti-aging through the inhibition of mTOR is quite complicated. In fact, it is not only complicated but also sometimes looks like a paradox. In *C. elegans*, Mizunuma et al. reported that mTORC2 modulates longevity by activating SGK-1 in two pathways that affect lifespan oppositely. RICT-1/mTORC2 limits lifespan by directing SGK-1 to repress the intestinal stress response transcription factor SKN-1/Nrf. Furthermore, RCT-1/mTORC2 functions in SGK-1-mediated pathways in lifespan extension at lower temperatures. Thus, RICT-1/mTORC2 and SGK-1 can inhibit or accelerate aging, depending on their context of action [44]. 

There is growing recognition that life-extending procedures may have sex-differential effects on survival. Harrison found that rapamycin led to an increase of 14% for females and 9% for males in extending lifespan [45]. As mentioned earlier, three cases also demonstrated that only female mice, but not male mice, enjoyed lifespan extension when their mTOR pathways were perturbed [4,5,6]. It looks like only the females showed higher life expectancy when treated by mTORC1 inhibition. Interestingly, in one case, it was shown that females, rather than males, did not enjoy these metabolic benefits when they were treated with ACA or 17aE2 [7], even though they concluded that male-specific metabolic improvements were associated with enhanced hepatic mTORC2 signaling, increased Akt activity, and phosphorylation of FOXO1a. Dominick et al. constructed Snell dwarf and global GH receptor (GHR) gene-disrupted mice (*ghr*^−/−^), who had lower mTORC1 activity in liver, muscle, heart, and kidney tissues [46]. Surprisingly, mTORC2 activity was higher in fasted Snell and *ghr*^−/−^ mice than in littermate controls in all four tissues tested. However, a systemic or tissue-specific (brain, liver, or adipose tissue) lack of mTORC2 signaling impaired metabolic health and shortened lifespan in wild-type and long-lived mice.

Some evidence shows that the sustained activation of mTOR downregulates insulin signaling in insulin-responsive cells and reduces insulin secretion in beta cells, which contributes to insulin resistance. However, Vodenik et al. thought that mTOR also generates an inhibitory feedback loop on insulin receptor substrate (IRS) proteins [47]. Chronic treatment of rapamycin can severely impair glucose tolerance and insulin action [4]. Chronic treatment of rapamycin disrupts mTORC2, which is required for insulin-mediated inhibition of hepatic gluconeogenesis in vivo. Disruption of mTORC2 is an important mediator of the action of rapamycin in vivo. This is a key process involving many oncogenic proteins such as cyclin D1, c-Myc, Mcl-1, Snail, Akt, serum and glucocorticoid-induced kinase (SGK) and protein kinase C (PKC). In contrast to mTORC1, the biological functions of mTORC2, especially those related to the regulation of tumorigenesis rather than its functions in regulating the cytoskeleton and cell survival, are not fully understood. Teutonico et al. reported that tacrolimus was associated with a significant reduction in insulin sensitivity and with a defect in the compensatory β-cell response. Clinically, the switch to sirolimus, also known as rapamycin, increased a 30% incidence of impaired glucose tolerance and with four patients developing new-onset diabetes (NOD) [48]. Johnston et al. found that sirolimusis is independently associated with NOD [49].

SASP is partially regulated by mTOR, possibly through the regulation of IL1A translation or MAPKAPK2 signaling, and is suppressed while using mTOR inhibitors [50,51] or MAPK inhibitors [52]. Rapamycin and torin have also been reported to inhibit the anti-inflammatory effects of circulating glucocorticoids [53]. Transplant patients receiving mTORC1 inhibitors more than twice are likely to develop noninfectious fever, suggesting excess inflammation [54]. The pro-inflammatory effect of mTORC1 inhibition may be induced by high doses, whereas the anti-inflammatory inhibition of SASP may be achieved at low doses [24]. Although mTOR inhibition still has some disadvantages, it produces more advantages in fact. The effect of targeting mTOR for anti-aging therapy is irrefutable.

## 4. Cancer Therapy by Targeting mTOR

Cancer therapy by targeting mTORC1 signaling has been extensively studied over the past decades, and cancer therapy by targeting mTORC2 has received more and more focus recently. The situation in cancer therapy targeting mTOR is very complicated because the signal pathways always influence each other and coordinate with each other.

Studies on the tuberous sclerosis complex, a well-known negative regulator of mTORC1, provided direct evidence for mTORC1 in tumorigenesis. Feng et al. demonstrated that p53, a tumor suppressor, regulates the mTOR pathway through AMPK activation and the TSC1/TSC2 complex and regulates autophagy [55]. They concluded that the p53 and mTOR pathways could cross-talk and coordinately regulate cell growth, proliferation, and death. While the regulation of protein synthesis is largely attributed to mTORC1, mTORC2 regulates members of the AGC kinase family and is implicated in various diseases, including cancer and diabetes. It was reported that growth factors activate mTORC2 through PI3K signaling [56,57,58]. Recent evidence suggests that activated mTORC2 is physically associated with ribosomes, and insulin-stimulated PI3K signaling promotes the binding between mTORC2 and ribosomes, suggesting that ribosomes are involved in the activation of mTORC2 [59]. mTORC2 promotes cell survival through Akt and mediates the organization of the actin cytoskeleton [60,61]. It was found that ribosomes were upstream of TORC2, and mNIP7 deletion inhibited TORC2 kinase activity in vitro and in vivo. These data suggested that the disruption of the mTORC2-ribosome supercomplex selectively induced apoptosis in PTEN-deficient melanoma cells. The observation that mTORC2 was required for tumor progression in some cancers suggested that myc and ribosomes may promote tumorigenicity by stimulating mTORC2 and its downstream effector, Akt [62,63,64]. Moreover, it was suggested that TORC1 indirectly controls TORC2 via the activation of ribosome biogenesis and the inhibition of autophagy-induced ribosome turnover. Sarbassov et al. also reported that long-term rapamycin treatment indirectly inhibits mTORC2 [65]. Thus, blocking the mTORC2-ribosome interaction could be an effective strategy for the treatment of melanoma, colon cancer, and possibly other cancers. Gulhati et al. showed that rapamycin significantly decreased the proliferation of certain sporadic colorectal cancer (CRC) cell lines (rapamycin sensitive), whereas other cell lines were resistant to its effects (rapamycin resistant). siRNA-mediated knockdown of Rictor, one of the mTORC2 subunits, significantly decreased the proliferation of both rapamycin-sensitive and rapamycin-resistant CRC cells. Furthermore, the stable downregulation of mTORC1 and mTORC2 reduced the proliferation and cell cycle progression in rapamycin-sensitive and rapamycin-resistant CRCs, whereas only the downregulation of mTORC2 increased apoptosis in rapamycin-resistant CRCs [63]. These suggest that selectively targeting mTORC1 and mTORC2 may be a new therapeutic strategy for the treatment of colorectal cancer.

Cancer cells proliferate faster than normal cells, which means that they need more energy, lipids, amino acids, nucleotides, and so on. mTORC1 regulates aerobic glycolysis by increasing the translation of hypoxia-inducible factor (HIF)-1α, a transcription factor that drives the expression of multiple glycolytic enzymes [66,67]. mTORC1 phosphorylates Lipin1 and S6K1, upregulates the synthesis of lipids from glycolysis-derived intermediates and activates the transcription factor sterol regulatory element-binding factor (SREBP)-1 to drive the transcription of lipogenesis-related genes [68,69]. Ricoult et al. found that SREBPs support oncogene-induced cell proliferation and growth, and the aberrant activation of mTORC1 in human breast cancer promotes adipogenesis programs through the activation of SREBP and its gene targets [70]. Rapid DNA replication, such as the synthesis of purines and pyrimidines, is also necessary for cancer cells, which is mediated by the phosphorylation of S6K1 through mTORC1 [71,72,73]. These suggest that the targeted inhibition of the mTORC1 pathway is a viable way to block cancer cell growth. 

Low ATP/high AMP levels activate AMP kinase (AMPK), an indirect mTORC1 inhibitor that acts by promoting the activity of the TSC1/TSC2 complex [74]. The accumulation of AMP overrides growth factor signaling and prevents cell proliferation in the absence of an adequate energy supply. Studies of prostate cancer have shown that the most common targets of increased translation through mTOR are those involved in invasion, metastasis, and protein synthesis [75,76]. mTOR is deregulated in nearly 100% of advanced human prostate cancers, and genetic studies in mouse models suggest that mTOR is overactivated during prostate cancer initiation [62,75,77,78,79]. Once activated, mTORC1 phosphorylates elongation initiation factor (eIF)-4E-binding protein 1 (4EBP1) and ribosomal protein S6 kinase 1 (S6K1), which promote translation. Increased cap-dependent translation caused by aberrant mTORC1 activation leads to increased cell size and proliferation, which are two common hallmarks of cancer [80,81]. Liu et al. found that mutations of key Sin1 residues mediating phosphatidylinositol (3,4,5)-triphosphate (PIP3) interactions inactivate mTORC2, whereas patient-derived mutations in the Sin1-PH domain pathologically increased mTORC2 activity, promoting cell growth and tumor formation [82]. Now, AKT has been reported as one of the most frequently highly activated proteins in cancer. AKT integrates signals from PI3K/mTORC2 and PI3K/PDK1 to promote cell growth and survival. Guertin et al. reported that the partial loss of mTORC2 activity extends lifespan and protects mice against prostate cancer in *pten*^+/−^ mice [62]. mTORC2 promotes GBM growth in glucose through a Myc-dependent, AKT-independent signaling pathway, and mTORC2 regulates c-Myc levels and glycolysis through FoxO acetylation [83]. These results demonstrate the important role of mTORC2 in cancer metabolism and suggest that the inhibition of mTORC2 may be useful in the treatment of cancer patients. The disruption of the *rictor* gene blocks mTORC2-dependent ductal branching and reduces mammary epithelial cell viability, invasion, and survival [84]. In total, 70% of gliomas contain high levels of activated Akt, which is associated with mTORC2 activity [64]. Morrison et al. found that mTORC2 signaling drives AKT-dependent tumor progression in HER2-amplified breast cancer, and mTORC1/2 dual kinase inhibitors reduce the survival of AKT-dependent HER2-amplified cells [85].

The PI3K/Akt/mTOR axis is implicated in idiopathic pulmonary fibrosis (IPF), which is considered a risk factor for lung cancer [86]. TGF-β1 can also induce the phosphorylation of mTORC1 substrates, including p70 S6K (Thr389) and 4E-BP1 (Thr37/46, Ser65), as well as mTORC2 substrates, such as Akt (Ser473) and SGK1 (Thr346). Woodcock et al. demonstrated that TGF-β1 activates the Smad3 pathway, which mainly activates COL1A1 mRNA expression and the mTORC1/4E-BP1 axis, further stimulating collagen synthesis in human lung fibroblasts [87]. Meanwhile, TGF-β1-induced collagen synthesis is independent of canonical PI3K/Akt signaling, and targeting this axis may be a potential anti-fibrotic strategy in multiple fibrotic conditions. However, Junpaparp et al. found that everolimus, an mTOR inhibitor, can induce severe pulmonary toxicity with diffuse alveolar hemorrhage [88]. Malouf et al. also found that everolimus use was associated with more rapid disease progression in patients with idiopathic pulmonary fibrosis [89]. Research on targeting mTORC1 signaling in the context of IPF still continues. However, the rapalog everolimus failed to show any benefit in IPF clinical trials and was found to be partially associated with the downregulation of mTORC2 activity after chronic treatment [4,65,89]. Although ATP-competitive mTOR inhibitor was shown to be highly effective in blocking collagen synthesis in live precision-cut IPF lung tissue slices, the long-term tolerability of dual mTORC1 and mTORC2 inhibition remains unknown. The risk–benefit ratio for chronic fibrotic disease remains to be determined [90].

Rapamycin exhibits antiangiogenic activity associated with the reduced production of vascular endothelial growth factor (VEGF) and significantly inhibits endothelial cell response to VEGF stimulation [91]. The pretreatment of PC-3 cells with the mTOR inhibitor rapamycin inhibited both HIF-1 accumulation and HIF-1-dependent transcription induced by hypoxia or CoCl2. These studies position mTOR as an upstream activator of HIF-1 function in cancer cells and suggest that the antitumor activity of rapamycin is mediated in part by inhibiting cellular responses to hypoxic stress [92]. VEGF inhibitors were more effective at blocking new blood vessel formation, and everolimus was more effective at reducing the viability of existing blood vessels [76]. Rapamycin inhibits endothelial Akt signaling, vascular alterations, tumor growth, and tumor vascular permeability. Akt signaling in the tumor vascular stroma is sensitive to rapamycin, suggesting that rapamycin may affect tumor growth partly by acting as an inhibitor of vascular Akt [93]. mTOR inhibitors exhibited increased efficacy as antiangiogenic agents when they were used in combination with radiotherapy [94]. The dual inhibition of the PI3K/mTOR pathway increases tumor radiosensitivity by normalizing tumor vasculature [95].

In tumor immunity, melanoma biospecimens after PD-1 treatment showed significantly reduced p-S6 expression compared with patient-matched pre-treatment biopsies, consistent with the findings in PD-1 antibody-treated melanoma cell lines [96]. These data suggest that a combination of immune checkpoint inhibitors and mTOR inhibitors may be more effective. In fact, many pharmaceutical companies are conducting clinical trials of combinations of PI3K/mTOR inhibitors and immune checkpoint inhibitors [97,98,99]. mTOR is required for Th1 and Th2 effector T cell differentiation. mTOR-deficient T cells fail to differentiate into Th1 or Th2 effector cells, implicating that both mTORC1 and mTORC2 in preventing the generation of regulatory T cells [100]. Component-active Akt suppresses Treg cell development, whereas rapamycin promotes their development at the expense of Th17 cell differentiation [101,102].

These data seem to suggest that mTORC1 or mTORC2 inhibition has benefits for cancer therapy, and dual mTORC1 and mTORC2 inhibition is better than either one in many ways.

## 5. The Disadvantage of Cancer Therapy by Targeting mTOR

In fact, targeting mTOR to treat cancer is also confusing in some ways. Pusapati et al. found that glycolysis-resistant ovarian cancer cells cultured with 2DG have upregulated mTORC1 activity and increased adipogenesis and nucleotide synthesis [103]. This suggests that mTORC1-mediated glycolysis may support cancer cells. However, in the absence of glycolysis, other mTORC1-mediated anabolic pathways still support cell proliferation. How these tumor cells acquire the necessary intermediates to shunt other metabolic pathways in the absence of glycolysis remains a question.

Palm et al. found that protein macropinocytosiscan also serve as an essential amino acid source, and the activation of mTORC1 suppresses proliferation when cells depend on extracellular proteins as a source of amino acids [104]. Recently, much evidence has shown that the PI3K pathway is frequently activated in response to oncogenic growth factor receptor signaling. Activating mutations in PIK3CA, mutations in Ras, or loss of PTEN lead to increased production of the second messenger PIP3. PIP3 directly recruits and activates mTORC2, and PI3K signaling also indirectly activates mTORC1, mainly through AKT. The activation of AKT occurs through mTORC2-mediated phosphorylation at Ser473 and PDK1-mediated phosphorylation at Thr308.

In tumor cells deficient in amino acids in culture and in poorly vascularized tumors in vivo, mTORC1 inhibition confers a growth advantage through the upregulation of macropinocytosis and the catabolism of phagocytic proteins [104]. The dual role of mTORC1 in tumor metabolism and growth requires further consideration, especially in patients treated with mTOR inhibitors. Nutrients and growth factors activate mTORC1 and inhibit autophagy through the phosphorylation of various autophagy-related proteins, such as ULK1, ATG13, AMBRA1, and ATG14L, and promote autophagy initiation and autophagosome nucleation [99]. In some cases, autophagy inhibits tumorigenesis; but in most cases, autophagy promotes tumorigenesis [105]. Autophagy was originally considered to be a tumor suppression mechanism, stemming from the early reports of monoallelic loss of the essential autophagy gene *atg6/becn1* in 40–75% of human prostate, breast, and ovarian cancers [106,107,108]. The p53 pathway is also a master regulator of cell fate decisions involved in DNA damage-induced apoptosis, senescence, or autophagy, especially during the G1/S phase. Gupta et al. found that DNA damage-induced autophagy by miR-16 at the G1/S checkpoint requires a functional p53 pathway. Interestingly, the AMPK/mTORC1/ULK1 pathway may be an alternative in p53-deficient cell lines such as HeLa, H1299, and PC-3 [109]. These highlighted the importance of the miR-16/p53/mTORC1 pathway for the inhibition of cell proliferation through autophagy. In some cell-based assays, autophagy inhibition promoted cancer cell growth, and *becn1* heterozygous mutant mice were prone to develop liver and lung tumors and lymphomas with prolonged latency [110,111]. However, autophagy is also upregulated in Ras-transformed cancer cells and promotes their growth, survival, tumorigenesis, invasion, and metastasis [112,113,114,115]. Human endothelial cells (ECs) under mTORC1 inhibition are resistant to apoptosis after removal of the inhibition and are hyper-responsive to renal cell carcinoma (RCC)-stimulated angiogenesis [116]. mTORC2 inactivation reduces EC migration more than Akt1- or mTORC1 inactivation. Dual mTORC1/2 inhibition or selective mTORC2 inactivation suppresses angiogenesis in RCC cells.

Rapalogs have some shortcomings in terms of cancer treatment efficacy. The inhibition of mTORC1 by rapamycin blocks the phosphorylation of S6K1, but the phosphorylation of 4EBP1 is weakly inhibited [117]. The inhibition of mTORC1 will increase PI3K/mTORC2/AKT signaling, which might improve cancer cell survival and result in faster cell growth [118,119]. mTOR causes self-resistance, inducing resistance to various mTOR inhibitors acting on it [120]. Phase I clinical trial with PI3K/mTOR dual kinase inhibitor shows significant dose-limiting on-target toxicity, which may be due to these enzymes playing a key role in the homeostasis of healthy tissues and systemic metabolism [121,122].

## 6. Conclusions

**Figure 2:**
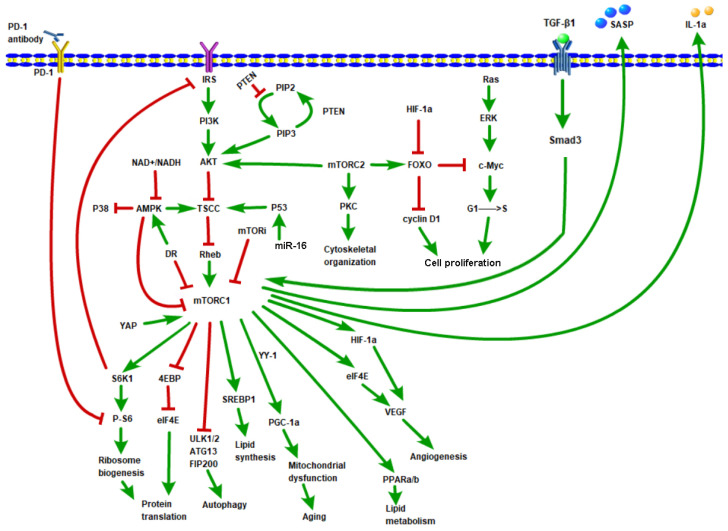
An overview of mTOR pathway. Green arrow means promoting effect, red T-shaped line represents the inhibitory effect.

Cellular senescence, an irreversible cell cycle exit, is traditionally viewed as an important tumor suppression mechanism [14]. However, Milanovic et al. challenged this traditional view, showing that senescence can promote cancer severity and tumor aggressiveness [123,124]. Yasuda et al. also showed the relationship between SASP and cancer. In a mouse model, the pro-inflammatory cytokine-driven downregulation of EZH2 maintains SASP by demethylating H3K27me3 marks and promotes peritoneal tumor formation in gastric cancer (GC) via JAK/STAT3 signaling [125]. These findings suggest that targeting aging could be used in cancer treatment. This means that it is possible to simultaneously delay aging and treat cancer by targeting mTOR. Although the mechanism of mTOR inhibition for anti-aging or cancer therapy is complicated (Figure 2) and sometimes confusing, it is believed that mTOR inhibition is generally beneficial for anti-aging and anti-cancer. Rapalogs generally lead to disease stabilization rather than regression, consistent with the idea that mTORC1 is a driver of cell proliferation rather than cell survival [76]. The combination of mTOR inhibitors and other anti-cancer therapies, such as NK cells, Car-T cells, PD-1 inhibitors, and PD-L1 inhibitors (Table 1), could be considered. In fact, many pharmaceutical companies have been working toward this direction. 

## Figures and Tables

**Table 1:** Clinical investigations evaluating the efficacy of mTOR inhibitors in a combination for the treatment of HNSCC.

Drug	Population	Phase	Status	Trail Identifier
Temsirolimus + Er	Advanced HNSCC	Phase II	Terminated	NCT01009203
Temsirolimus + Cet	R/M HNSCC	Phase II	Completed	NCT01256385
Temsirolimus + Cis, Cet	R/M HNSCC	Phase I/II	Terminated	NCT01015664
Temsirolimus + Pac, Car	R/M HNSCC	Phase I/II	Completed	NCT01016769
Everolimus + Cis Rad	HNSCC patients	Phase I	Terminated	NCT01057277
Everolimus + Cis IMRT	HNSCC patients	Phase I	Completed	NCT00858663
Everolimus + Er Rad	R/M HNSCC	Phase I	Withdrawn	NCT01332279
Everolimus + Doc, Cis	HNSCC patients	Phase I	Completed	NCT00935961

Abbreviations: Er, erlotinib; Cet, cetuximab; Cis, cisplatin; Pac, paclitaxel; Car, carboplatin; Rad, radiation; IMRT, intensity-modulated; Doc, docetaxel; R/M, recurrent or metastatic; HNSCC, head and neck squamous cell carcinomas.

## Data Availability

No new data were created.
